# Single-molecule tracking of PprI in *D. radiodurans* without interference of autoblinking

**DOI:** 10.3389/fmicb.2023.1256711

**Published:** 2023-11-02

**Authors:** Fanfan Zhai, Li Hao, Xiaomin Chen, Ting Jiang, Qianhong Guo, Liping Xie, Ying Ma, Xiaobo Du, Zhiqin Zheng, Kun Chen, Jun Fan

**Affiliations:** ^1^School of Life Sciences and Engineering, Southwest University of Science and Technology, Mianyang, Sichuan, China; ^2^Institute of Fundamental and Frontier Sciences, University of Electronic Science and Technology of China, Chengdu, Sichuan, China; ^3^NHC Key Laboratory of Nuclear Technology Medical Transformation (Mianyang Central Hospital), Mianyang, Sichuan, China; ^4^School of Biological Engineering and Wuliangye Liquor, Sichuan University of Science and Engineering, Yibin, Sichuan, China; ^5^School of Optoelectronic Science and Engineering, University of Electronic Science and Technology of China, Chengdu, Sichuan, China; ^6^Yangtze Delta Region Institute (Huzhou), University of Electronic Science and Technology of China, Huzhou, Zhejiang, China

**Keywords:** single-molecule tracking, PprI protein, mMaple3, autoblinking, *Deinococcus radiodurans*

## Abstract

Autoblinking is a widespread phenomenon and exhibits high level of intensity in some bacteria. In *Deinococcus radiodurans* (*D. radiodurans*), strong autoblinking was found to be indistinguishable from PAmCherry and greatly prevented single-molecule tracking of proteins of interest. Here we employed the bright photoswitchable fluorescent protein mMaple3 to label PprI, one essential DNA repair factor, and characterized systematically the fluorescence intensity and bleaching kinetics of both autoblinking and PprI-mMaple3 molecules within cells grown under three different conditions. Under minimal media, we can largely separate autoblinking from mMaple3 molecules and perform reliably single-molecule tracking of PprI in *D. radiodurans*, by means of applying signal-to-noise ratio and constraining the minimal length for linking the trajectories. We observed three states of PprI molecules, which bear different subcellular localizations and distinct functionalities. Our strategy provides a useful means to study the dynamics and distributions of proteins of interest in bacterial cells with high level of autoblinking.

## 1. Introduction

Fluorescence microscopy and related labeling strategies have changed the way how we study biological systems, yet lacks resolution at a scale comparable to biomacromolecules due to the diffraction of light. Single-Molecule Localization super-resolution Microscopy (SMLM), developed in the past two decades, has allowed scientists to visualize the structural organizations of cells at molecular resolution with high specificity (Stracy et al., 2015, 2016; Zawadzki et al., 2015). In this technique, super-resolution images are reconstructed by random photoactivation of fluorophores and subsequent localization of individual fluorescently labeled molecules (Betzig et al., 2006; Hess et al., 2006; Rust et al., 2006). Successful SMLM experiments require that the excited fluorophores are separated in space (Xie et al., 2008; Gahlmann and Moerner, 2014) and that a sufficient number of locations are accumulated over time to satisfy the Nyquist criterion for spatial resolution (Coltharp et al., 2014). Among the most prominent strategies of SMLM, Photoactivated Localization Microscopy (PALM) (Betzig et al., 2006; Hess et al., 2006; Nickerson et al., 2014) overcomes this problem by making use of photoactivatable or photoconvertible fluorescent proteins, such as mEos3.2 (Baldering et al., 2019), PAmCherry (Subach et al., 2009), or mMaple3 (Wang et al., 2014; Kaberniuk et al., 2018). UV light at low power level can activate a small subset of proteins to ensure that, at a time, there are very few emitting (photoactivated) molecules that switch reversibly between the photoactivated and dark states (a phenomenon also known as “blinking”) (Lee et al., 2012) and eventually bleach.

Combining single-molecule tracking (SMT) with PALM further provides direct access to the localizations, motions and interactions of individual proteins in cells (Manley et al., 2008), allowing a much broader range of biological questions to be investigated. Recent studies by single-molecule tracking of multiple DNA-binding proteins in live *E. coli.* cells have identified a general target search mechanism for DNA-binding proteins to accurately locate their target sites (Stracy et al., 2021). Single-molecule fluorescence microscopy has also been used to trace the dynamics of fluorescently tagged zinc-dependent transcription factors (TFs) in response to intracellular perturbations of unstable zinc, suggesting that there are transcription factors sensitive to the kinetics of zinc, revealing a potential new mechanism of transcriptional regulation (Damon et al., 2022).

Realizing the full potential of PALM and SMT relies on the accurate localization of each target fluorophore (Bohrer et al., 2021) without disturbance of spurious signals from autofluorescence. However, the recently reported auto-fluorescent spots in bacterial imaging place substantial interference on unbiased single-molecule localization, where such spurious fluorescent spots were found to be difficult to bleach completely in unlabeled cells, and thus likely indistinguishable from target fluorophores with similar blinking behavior (Liao et al., 2015; Tuson et al., 2016; Garza de Leon et al., 2017; Floc’h et al., 2018). This phenomenon is possibly associated with the transient and reversible binding of endogenous molecules in the cell or growth medium to the lipid bilayers on the plasma membrane, where their auto-fluorescence would be potentially enhanced (Floc’h et al., 2018). It is described as “autoblinking” (Floc’h et al., 2018) contrary to the real blinking behavior of exogenous fluorophores. Autoblinking is a common problem and presents to varying degrees in both gram-positive and gram-negative bacteria (Floc’h et al., 2018). For instance, the autoblinking level of *Escherichia coli* is low (Taniguchi et al., 2010; Floc’h et al., 2018), while *Bacillus subtilis* and *Deinococcus radiodurans* (*D. radiodurans*) exhibit much higher levels of autoblinking, with comparable brightness to the commonly used fluorescent proteins (FPs), e.g., PAmCherry (Liao et al., 2015; Tuson et al., 2016; Garza de Leon et al., 2017; Floc’h et al., 2018). This creates challenges for single-molecule imaging of low-copy-number proteins in bacterial cells (Tuson et al., 2016).

*Deinococcus radiodurans*, one of the most radioresistant organisms on Earth, possesses an extraordinary antioxidant system (Daly et al., 2010; Alekseev et al., 2020) and DNA repair capacity (Cox and Battista, 2005; Slade et al., 2009; Alekseev et al., 2020). Understanding DNA repair mechanisms at the single-molecule level with high spatiotemporal resolution can help answer crucial questions related to DNA damage response, and potentially benefit cancer therapy. However, high level of autoblinking in *D. radiodurans* cells significantly precludes single-molecule tracking of key proteins in DNA repair pathways, e.g., PprI, one of the central regulator and inducer proteins in DNA repair and transcription regulation. Thus, it is essential to resolve the influence of spurious fluorescent spots from autoblinking in single-molecule assays (Floc’h et al., 2018).

Here we show that using a bright photoconvertible fluorescent protein, mMaple3, PprI can be unambiguously localized, tracked, and analyzed at the single-molecule level with negligible autoblinking interference in *D. radiodurans* cells. We first minimize the spurious fluorescent spots by optimizing the growth/imaging medium, and then quantify their characteristics in fluorescence intensity, bleaching and recovery kinetics. We thus demonstrate a much shorter duration-frames in autoblinking and enable the differentiation from mMaple3. Single-molecule tracking reveals the significant differences in the mobility of fluorophores at nanoscale between autoblinking and PprI-mMaple3 molecules, further eliminating the interference on diffusing molecules. Together, the prospects of imaging proteins of interest in *D. radiodurans* cells and performing single-molecule tracking without the influence of spurious autoblinking provide exciting perspectives on the molecular mechanism of DNA repair.

## 2. Materials and methods

### 2.1. Strain construction

**FIGURE 1:**
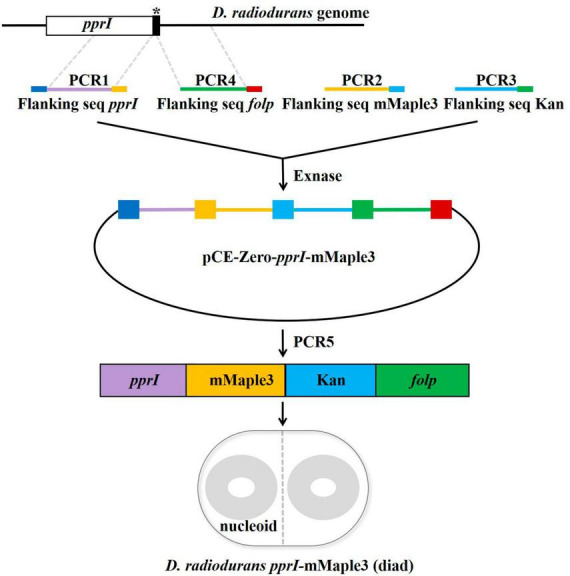
Construction strategy of the switchable fluorescent protein mMaple3 fused to the C-terminus of the *pprI* gene encoding PprI. The primers used for the insert are designed with a 20 bp homologous sequence at the 5′ end (marked with dark blue, yellow, light blue, green, and red in the picture), and the linearized vector and the insert are connected by the principle of seamless cloning and recombination. The linearized vector and insert are mixed in proportion, ligated under the catalysis of Exnase, amplified the ligated fragment and transformed into *Deinococcus radiodurans*-competent cells.

The C-terminus of PprI was labeled with the photoconvertible fluorescent protein mMaple3 (a gift from Stephanie Weber Lab) using homologous recombination (Manko et al., 2020; Figure 1 and Supplementary Table 1). Four PCR fragments were ligated by ClonExpress^®^ Ultra One Step Cloning Kit (Vazyme Biotech Co., Ltd., Nanjing), and then chemically transformed into *D. radiodurans* competent cells and selected by 20 μg/mL kanamycin (Sangon Biotech, Shanghai) for positive insertions. Based on multi-genomic *D. radiodurans* (Cox and Battista, 2005), the transformed colonies were further screened twice consecutively with selective medium containing 30 and 40 μg/mL kanamycin. The successful clones were confirmed by sequencing that mMaple3 and kanamycin resistance fragments have been integrated into the genome. The same method was used to construct a *D. radiodurans* strain in which the PprI protein was tagged with PAmCherry *in situ*.

For *E. coli*, the sequence of mMaple3 was inserted into the pET-28a vector using restriction enzyme (EcoRI/HindIII) (a gift from Jiang Shijie’s lab at Southwest University of Science and Technology) and then transformed into DH5α competent cells (Shanghai Weidi Biotechnology Co., Ltd, Shanghai) by heat shock method (Froger and Hall, 2007). Finally, the successful reconstituted vector was transformed into BL21 (DE3, Shanghai Weidi Biotechnology Co., Ltd, Shanghai) competent cells for mMaple3 expression.

### 2.2. SYBR Gold staining and sample preparation for microscopy

*Deinococcus radiodurans* strains were streaked onto TGY plates (10 g/L tryptone, 1 g/L glucose, 5 g/L yeast extract, 15 g/L ager) with kanamycin (20–40 μg/mL). Individual colonies were inoculated into Tryptone Glucose Yeast Agar Medium (TGY) or minimal medium (MM) (Venkateswaran et al., 2000; Supplementary Table 2) and grown at 30°C for 24 h, then diluted 100-fold into new TGY or MM medium and grown to the prophase of logarithmic growth (pro-log, at OD_600_ 0.3–0.5). Glass coverslips (VWR^®^ Micro Cover Glasses, No. 1 Slides, 0.17 mm) for *D. radiodurans* imaging were calcined in a muffle furnace at 500°C for 30 min in order to remove any background particles that were fluorescent in nature. A total of 1% agarose pads were prepared by mixing 2% low fluorescence agarose (Bio-Rad, Berkeley, California) with 2 × of either TGY, phosphate buffer (PBS), or MM medium (Zawadzki et al., 2015). For bacteria stained with SYBR Gold, 1 μL of 1:100 prediluted SYBR Gold was pipetted into 999 μL of MM liquid medium to make 1:10^5^ SYBR Gold culturing medium, in which the centrifuged bacteria were resuspended. The bacteria imaged on the PBS pad were washed six times with TGY-grown cells in PBS solution (Floc’h et al., 2018). Agarose pads between two glass coverslips were used to immobilize the cells during imaging processes. For BL21 individual colonies were picked and plated in 2 mL of M9 glycerol (0.2%), cultured overnight at 37°C until the OD_600_ was 0.4–0.6, and then diluted in fresh M9 to grow to an OD_600_ of 0.1. For fixation, the cells were centrifuged and resuspended in 4% paraformaldehyde in PBS buffer (Aladdin, Shanghai) and shaken at 22°C for 45 min. Cells fixed in this way were washed with PBS and immobilized on agarose pads (Uphoff et al., 2013; Zawadzki et al., 2015; Stracy et al., 2016).

### 2.3. TIRF microscopy

Total internal reflection fluorescence (TIRF) microscopy is widely used in single-molecule imaging because it reduces the inevitable defocusing fluorescence problem in fluorescence imaging systems (Stracy et al., 2014). In TIRF illumination, the excitation beam is reflected at the interface between the coverslip and the imaging medium so that the resulting evanescent excitation extends only 150 nm into the sample (Paige et al., 2001). To image target proteins in bacteria, we employed the near-TIRF system, also called highly inclined and laminated optical sheet (HILO) microscopy, which provides thin excitation light at a shallow angle to the coverslip and illuminates a few microns into the sample (Tokunaga et al., 2008). The custom-built imaging system was implemented with a 405 nm laser (MDL-III-405 100 mW, CNI Laser Co. Ltd) for photoactivation of mMaple3-fused proteins and a 561 nm laser (OBIS 561 nm LS 80 mW, Coherent) for excitation on an inverted microscope platform (IX83, Olympus). The photoactivation rate was controlled by a low level of 405 nm laser at a power of 0.02–0.08 W/cm^2^. Typical excitation power was 2.7 mW with the 561 nm laser at the sample, corresponding to a power density of 0.075 kW/cm^2^ at the sample, which allowed adequate signal and minimal photobleaching of mMaple3. While a high excitation power of 23.0 mW was used to remove the pre-existing fluorescence molecules prior to the photoactivation. A total of 405 nm and 561 nm lasers were collinearly combined and focused at the back focal plane of an oil-immersion objective lens (UPLAPO100XO, Olympus) through a dichroic mirror (ZT405/488/561rpc, Chroma). Fluorescence emission was filtered by a multiple band filter (ZET405/488/561x, Chroma) and an additional long-pass filter (ET575lp, Chroma) in front of an EMCCD (IXON-L-897, 512 × 512 pixels; Andor). An LED light source and a spotter (M660L4-C1, Thorlabs) were implemented for bright-field images. All the images were acquired by the EMCCD camera with a pixel size of 160 nm after a 100X objective (UPLAPO100XO, Olympus). Sample position and focus were controlled by a motorized piezoelectric stage, z-motor objective mount, and autofocus system (MS-2000, PZ-2000FT, CRISP, ASI Imaging). For single-molecule tracking experiments, an exposure time of 15.7 ms/frame was used throughout this article (unless otherwise specified).

### 2.4. Single-molecule data acquisition

Prior to the acquisition of single-molecule data, the pre-existing autofluorescence signal as well as mMaple3 fluorescent signal and autoblinking molecules in *D. radiodurans* cell were pre-bleached using 561 nm laser with high excitation power (23.0 mW). mMaple3 proteins were then repeatedly activated using a continuous low 405 nm and excited with a low 561 nm activation power (2.7 mW) to ensure that the single-molecule data was collected from freshly activated mMaple3 proteins by the 405 nm laser. Over 30,000 frames were typically recorded until no new activations were observed to acquire the vast majority of PprI molecules. For SYBR Gold-stained DR-PprI-mMaple3 bacteria, the images of nucleoid were acquired by briefly turning on 0.8 mW of 488 nm laser after locating the cells under bright field. The PprI-mMaple3 molecules were then imaged again as described above, so that co-localization of the nucleoid with PprI-mMaple3 molecules could be analyzed at single-molecule level. For the DR-PprI-PAmCherry strain, the same imaging conditions as for the DR-PprI-mMaple3 strain were used, to quantitatively compare the characteristics of mMaple3 and PAmCherry.

### 2.5. Image analysis

**FIGURE 2:**
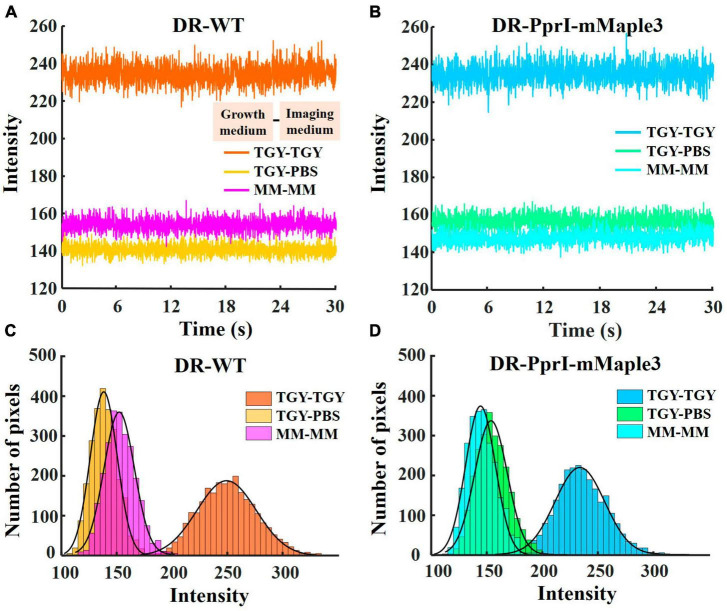
Background intensity under TGY, PBS and MM imaging medium. Time traces of background intensity when imaging DR-WT **(A)**, DR-PprI-mMaple3 **(B)** under TGY, PBS and MM (intensity as mean value from 5 regions of 4 × 4 pixels for 2000 frames for a total of 30 s, 1 frame equals to 0.0157 s). Histograms of background intensity when imaging DR-WT **(C)** and DR-PprI-mMaple3 **(D)** in TGY, PBS and MM (averaged from 4 regions of 50 × 50 pixels).

To analyze the background values, 5 background regions of 4 × 4 pixels without cells were selected randomly from the whole field of view of 256 × 256 pixels in the TGY, PBS, and MM environments for both wild-type (DR-WT, without introducing any extrinsic fluorescent markers) and fluorescently labeled *D. radiodurans* (DR-PprI-mMaple3), respectively (Supplementary Figure 1). The Measure Stack function in ImageJ was used to obtain the average intensity values of the intercepted 2000 frames (1 frame equals to approximately 0.015 s, total 30 s), and the Plot function was used in Matlab to draw the time traces of the background intensity changing with time (Figures 2A, B). Four regions of 50 × 50 pixels were selected from one specific frame for each imaging condition (Supplementary Figure 2). Background distributions in four regions were separately analyzed in each case (see Supplementary Figure 4). Then, the background values of the four regions were pooled together to obtain the histograms (Figures 2C, D). These six datasets were split into two groups based on strain type, and histograms of corresponding background intensities as a function of pixels number were plotted using Matlab (Figures 2C, D).

**FIGURE 3:**
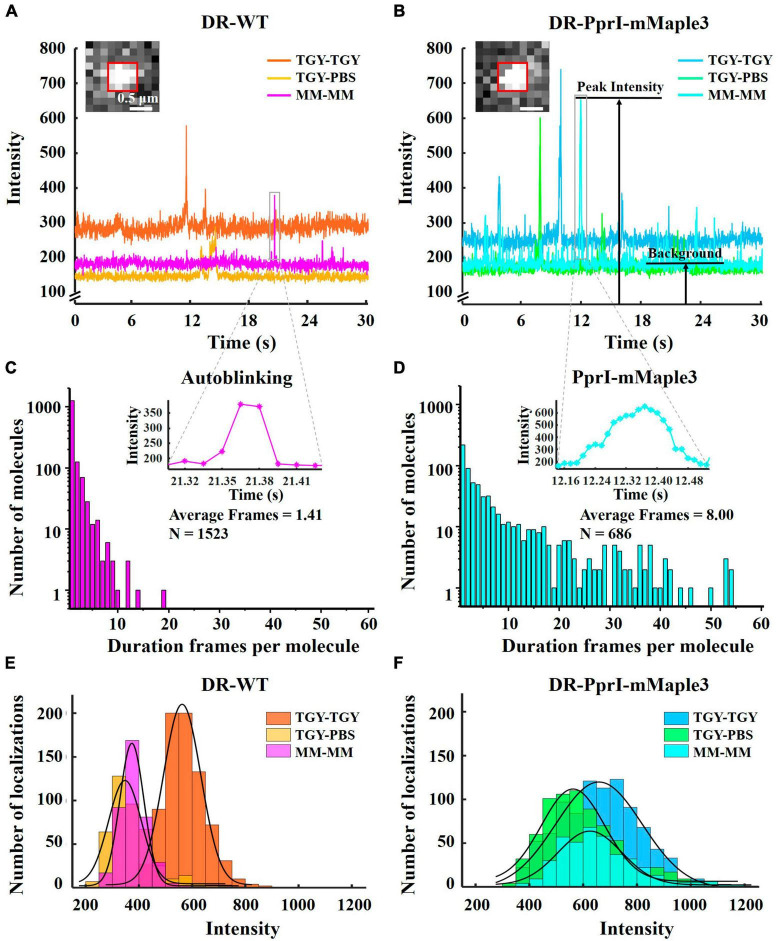
Fluorescence intensity and duration frames of autoblinking and PprI-mMaple3 molecules. **(A,B)** The intensity time traces of autoblinking of DR-WT and PprI-mMaple3 molecule of DR-PprI-mMaple3 bacteria in TGY, PBS and MM. **(C,D)** Histogram of duration frames per fluorescence molecule for autoblinking and PprI-mMaple3 molecules in the MM, the **top** **right** figure shows a magnified view of the fluorescent signal in **(A,B)**. **(E,F)** Histograms of autoblinking and PprI-mMaple3 molecules intensity when imaging DR-WT **(E)** and DR-PprI-mMaple3 **(F)** in TGY, PBS and MM. Scale bar: 0.5 μm in **(A,B)**.

To analyze the intensity time traces of autoblinking and mMaple3 molecules, 4 × 4 pixels regions with fluorescence signal (Supplementary Figure 3) were cropped from 2000 frames (1 frame equals to approximately 0.015 s, total 30 s) of 256 × 256 pixels in TGY, PBS, and MM environments for both DR-WT and DR-PprI-mMaple3, respectively. The time traces of the fluorescence intensity were plotted using the Plot function in Matlab (Figures 3A, B). To analyze the fluorescence intensity of autoblinking and PprI-mMaple3 molecules under different imaging environments, over 20 fluorescent signal molecules were selected in the above original video, and the Duplicate function in ImageJ was used to intercept the fluorescence signal frames from activation to irreversible bleaching (4 × 4 pixels). Use the Measure Stack function to extract the maximum signal strength value of each frame. The histogram function of Matlab was used to draw the corresponding fluorescence signal intensity normal distribution histogram (Figures 3E, F). Both Figures 2, 3A, B, E, F were analyzed from the same batch of experimental data with identical imaging conditions.

In the video of DR-PprI-mMaple3 bacteria acquired under MM imaging environment, 40 regions of 4 × 4 pixels were selected at the cell membrane or cell septum, and the average intensity of each 4 × 4 pixels was extracted using ImageJ. For autoblinking, the time traces of intensity were used to determine the background values. The number of duration frames whose average intensity is greater than the background value is calculated by Matlab and one frame is allowed to be lost. The hist function is used to obtain the histogram of the duration frame of each autoblinking molecule (Figure 3C). The histogram of the number of duration frames of the PprI-mMaple3 molecule was obtained in the same way by selecting 100 regions of 4 × 4 pixels of the fluorescence signal in the cell (Figure 3D).

### 2.6. Molecule localization and tracking

Combining the single-molecule tracking technology with the PALM-centered photoactivation strategy, densely localized molecules can be imaged and tracked (Manley et al., 2008). Single-molecules are sparsely photoactivated and imaged for multiple frames until they are irreversibly photobleaching. Based on the blinking properties of mMaple3 molecules, we used a one-frame memory parameter to account for the transient disappearance of fluorophore images within the trajectory due to blinking or missed localizations (Uphoff et al., 2014; Stracy et al., 2015). Trajectories were created by connecting the molecular localizations appearing on consecutive frames of images. When other molecules are randomly activated, the process is repeated until all the molecules of interest are imaged. Using a lower excitation intensity allows the molecules to be tracked for a longer duration (Stracy et al., 2014).

Single-molecule tracking analysis was carried out using ImageJ and custom-written Matlab software (Crocker and Grier, 1996). The outline and midline of the cells were obtained by segmenting the cells with edge detection using Microbe Tracker software on bright field images (Sliusarenko et al., 2011), and the *x*-axis was defined as the short axis of the cells and the *y*-axis as the long axis of the cells to determine the position of the molecular trajectories relative to the midline (Sliusarenko et al., 2011). Molecular trajectories were plotted using Matlab, and the diffusion rates of molecules were color-coded. We followed the molecules only within the boundaries of cells and identified the fluorophore images for localization through band-pass filtering and applying intensity thresholds to each frame of the movie. If the cell contains bright spots that are not bleached during 561 nm pre-bleaching, or if the cell has an abnormally high fluorescence background, it is excluded from the analysis. PprI-mMaple3 molecules and autoblinking molecules were detected, fit to symmetrical 2D Gaussian point spread functions (PSFs), and tracked using Matlab. A custom script was applied to create regions of interest (ROIs) based on the cell masks generated by Microbe Tracker. All of the localizations within each ROI were then tracked and analyzed separately. Tracks were linked to trajectories if they appeared in successive frames in a 3-pixels (0.48 μm) window. Moreover, we used a one-frame memory parameter to minimize the transient loss of fluorophore images within the trajectory due to blinking or loss of positioning.

### 2.7. Fitting D* distributions

We distinguished immobile and diffusing proteins by calculating an apparent diffusion coefficient D* = MSD/(4Δt) from the mean-squared displacement (MSD) and taking only trajectories with at least 4 steps and Δt = 15.7 ms, Δt is the lag times. For each measurement, the σ_loc_ of localization precision is expressed as a positive offset (Michalet and Berglund, 2012; Garza de Leon et al., 2017) in the D* value of the σ_loc_^2^/Δt. We used the same D* value of immobile molecules to constrain one species and fit the data well for a second and/or third unconstrained species (Supplementary Figure 5).

In order to obtain the localization error, 100 mMaple3 molecules were measured in the fixed *E. coli* cells expressing the plasmid carrying mMaple3. Each molecule presented a cluster of localizations owing to continuous acquisition of the same molecule. Localizations from 100 clusters (each containing >8 localizations) were aligned by their center of mass to generate the 2D presentation of the localization distribution. Histograms of distribution in x and y were fit to Gaussian functions, and the resultant s.d. (σx and σy) was shown. This data evaluated the single-molecule localization accuracy (Supplementary Figure 6) and was used to distinguish between immobile and diffusing PprI-mMaple3 molecules.

As mentioned elsewhere (Vrljic et al., 2002; Stracy et al., 2015, 2016; Garza de Leon et al., 2017), we obtained the diffusion coefficient D_1_* by fitting the probability density of the sample D* distribution with the analytical equation of D_1_*. The analytical expression of the single-mode case is:


$$f_{D^{*}}\,\left(x;D_{1}^{*}\right)=\frac{{\left(4/D_{1}^{*}\right)}^{4}\,x^{3}\,e^{-4\,x_{/}\,D_{1}^{*}}}{6},$$

where x is the empirical D* data. It is hypothesized that there may be three species of PprI with different rates of movement: molecules that diffuse rapidly into the cytoplasm, slowly moving DNA-searching molecules, and PprI that binds specifically to DNA. As a result, we introduced a third.


$$f_{D^{*}}\,\left(x;D_{1}^{*},D_{2}^{*},D_{3}^{*},A_{1},A_{2},A_{3}\right)=\frac{A_{1}\,{\left(4/D_{1}^{*}\right)}^{4}\,x^{3}\,e^{-4\,x_{/}\,D_{1}^{*}}}{6}+$$

$$\frac{A_{2}\,{\left(4/D_{2}^{*}\right)}^{4}\,x^{3}\,e^{-4\,x_{/}\,D_{2}^{*}}}{6}+\frac{A_{3}\,{\left(4/D_{3}^{*}\right)}^{4}\,x^{3}\,e^{-4\,x_{/}\,D_{3}^{*}}}{6},$$

where D_1_*, D_2_*, and D_3_* are the diffusion coefficients for the three different species, A_1_, A_2_, and A_3_ are the fractions of the molecules found in each of the states, and A_1_+A_2_+A_3_ = 1, respectively.

## 3. Results

### 3.1. Minimal medium greatly suppresses the background intensity in *D. radiodurans* cells

Culturing media greatly influences the growth status of bacteria, and it has been shown that the rich growth media could contribute to autoblinking (Floc’h et al., 2018). Although photoconvertible fluorescent protein has been widely applied in single-molecule tracking with good photophysical characteristics and optimal cell compatibility, PAmCherry had been identified as a non-ideal fluorescent probe for *D. radiodurans* in previous studies (Liao et al., 2015; Garza de Leon et al., 2017; Floc’h et al., 2018). We employed another photoconvertible fluorescent protein, mMaple3 with better photostability and brightness (Wang et al., 2014), to label PprI protein (DR-PprI-mMaple3) in *D. radiodurans* (Figure 1). To verify the effect of imaging media on the detection of fluorescent proteins, we measured background intensity in wild-type *D. radiodurans* (DR-WT) and DR-PprI-mMaple3 cells under different imaging media using TIRF microscopy (Figures 2A, B). In TGY, the background intensities stand at the highest level for both DR-WT and DR-PprI-mMaple3, with the average of 234 ± 5.03 and 235 ± 5.11 a. u., respectively (Figures 2A, B). In comparison, the background intensity in PBS or MM were much lower, ∼150 a. u., which was dramatically different from that in TGY. For DR-WT, the background intensity of 141 ± 2.77 a. u. in PBS was slightly lower than that of 154 ± 3.25 a. u. in MM (Figure 2A); whereas in DR-PprI-mMaple3, the background intensity of 157 ± 3.30 a. u. in PBS was similar as that of 148 ± 3.15 a. u. in MM imaging medium (Figure 2B). The background intensities in PBS and MM showed no significant differences, which was independent of imaged strains, suggesting both washed cells and growing cells in MM, instead of TGY, were effective for reducing the background. In addition, the time traces of background intensities showed no visible drift, indicating a constant background with a reliable imaging system.

To verify whether the averaged background intensity of the entire field of view is independent of spatial locations, we randomly select 4 regions of 50 ± 50 pixels in one frame (Supplementary Figure 2). It can be found that the background levels are similar in different regions, demonstrating the uniformity of the spatial distribution of background (Supplementary Figure 4). Histograms of the spatial distribution (Figure 2) showed that the mean background intensity from DR-WT samples under TGY, PBS, and MM were 251 ± 26.78, 139 ± 12.17, and 153 ± 13.71 a. u. respectively (Figure 2C). And the mean background intensity of DR-PprI-mMaple3 in TGY, PBS, and MM were 235 ± 22.37, 155 ± 14.70, and 146 ± 13.37 a. u. (Figure 2D). These results suggested that the mean background intensity of spatial distribution was consistent with that of temporal distribution (Figures 2A, B). Furthermore, both strains growing in PBS and MM showed greatly reduced background, allowing a good imaging condition for acquiring single-molecule signals from *D. radiodurans*.

### 3.2. Enhanced fluorescence intensity and long-lasting duration of PprI-mMaple3 molecules

To investigate the brightness level and fluorescence stability of autoblinking molecules and PprI-mMaple3 molecules, we observed the fluorescence intensity trend of the two species from DR-WT and DR-PprI-mMaple3 samples in TGY, PBS and MM imaging medium for 2000 frames in the selected fluorescence region (Figures 3A, B). And we compared the fluorescence intensities of the two species in three imaging environments (Figures 3E, F, Supplementary Figure 3). Among them, the fluorescence signals in DR-WT bacteria were all autoblinking molecules, while the fluorescence signals in DR-PprI-mMaple3 bacteria might have been mixed with a small amount of autoblinking molecules. In DR-WT samples, the single-molecule autoblinking intensity was up to 572 ± 70.40 a. u. in TGY imaging medium, while it decreased to 372 ± 61.70 a. u. and 382 ± 45.02 a. u. in PBS and MM (Figure 3E), respectively (Supplementary Table 3). We further quantified the signal-to-background ratios (SBR), defined as the ratio of the peak intensity to the averaged baseline background of the same acquired region (Figure 3B). For DR-WT bacteria, the SBRs of autoblinking molecules in TGY, PBS, and MM were 2.00, 1.98, and 2.05 (Supplementary Table 3), respectively, indicating that the SBRs did not show much difference among 2000 selected frames from DR-WT cells (Figure 3A). In contrast, there was little difference in intensity of PprI-mMaple3 molecules in TGY, PBS, and MM, which were 664 ± 160.45 a. u., 606 ± 117.32 a. u., 661 ± 117.87 a. u. (Figure 3F and Supplementary Table 3), respectively, indicating that the fluorescence intensity of mMaple3 was unaffected by the bacterial growing conditions. However, SBRs of PprI-mMaple3 increased in PBS and MM imaging medium due to the reduced background noise (Figure 3B), which were 2.96, 3.51, and 3.58 (Supplementary Table 3), respectively. These results indicated that the mMaple3 molecules in PBS and MM imaging medium were brighter than autoblinking molecules, and the higher SBR made the fluorescence signal of PprI-mMaple3 more visible in PBS and MM imaging medium. However, PBS imaging medium with zero nutrition might affect *D. radiodurans* growth, leading to abnormal expression of PprI. Therefore, MM medium was considered as the ideal imaging medium for our single-molecule tracking experiments in *D. radiodurans* cells, and subsequent experiments in this paper were imaged in MM medium.

The SBRs and the number of localizations (Supplementary Table 4) at the same number of acquisition frames (14,654 frames) were compared between the PprI-mMaple3 molecules and the PprI-PAmCherry molecules using the same method. The result shows that mMaple3 provided a much larger number of PprI localizations, which was ∼4-fold higher than that of the PprI-PAmCherry molecules in the same imaging period. Comparing the SBRs of mMaple3, PAmCherry, and autoblinking, it is clear that the SBR of autoblinking is only slightly lower than that of PAmCherry, making these two hardly distinguishable.

To further evaluate the photostability of these two species, we calculated the duration frames of autoblinking (Figure 3C) and PprI-mMaple3 molecules (Figure 3D), respectively. The mean of duration frames was only 1.41 for autoblinking molecules (Figure 3C), while the average duration frames of PprI-mMaple3 molecules were 8, with the maximum reaching 54 frames (Figure 3D), showing a huge difference in duration frames between autoblinking and PprI-mMaple3 molecules. Consequently, the majority of autoblinking molecules (trajectories ∼4 steps) could be removed based on the duration frames of the fluorescent molecules (autoblinking molecules and mMaple3 molecules) during the data analysis.

### 3.3. The subcellular distribution and mobilities of autoblinking and PprI-mMaple3

**FIGURE 4:**
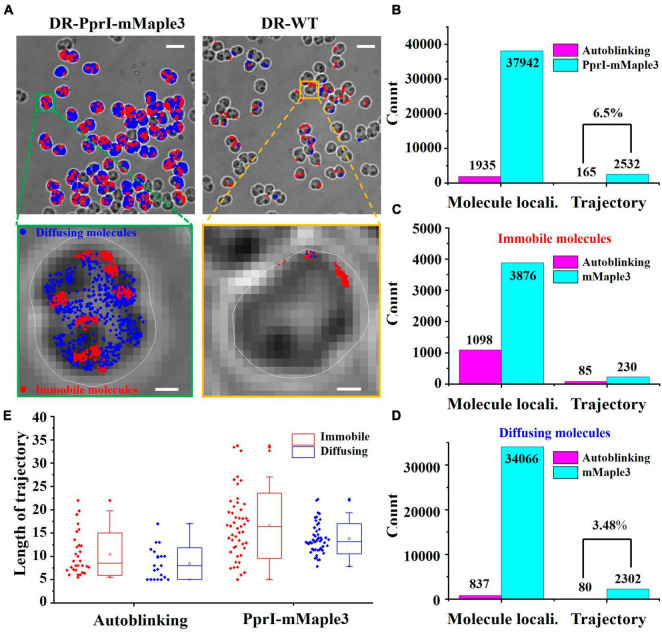
Verification of the differentiated species of autoblinking and PprI-mMaple3 molecules in DR cells. **(A)** Subcellular localizations of the PprI-mMaple3 and autoblinking molecules under the same imaging conditions. Scale bar: 0.5 μm. **(B–D)** Comparison of localizations and trajectories of autoblinking and PprI-mMaple3 molecules in terms of the entire population and distinguished immobile vs. diffusing molecules. **(E)** Trajectory lengths of immobile and diffusing molecules for autoblinking and the PprI-mMaple3 molecules.

Although the autoblinking molecules have lower signal intensity and shorter average duration frames compared to the PprI-mMaple3 molecules, these two parameters can remove the majority of the autoblinking molecules, and a tiny fraction of those ones with stronger signal intensity and longer duration frames still remain. In order to quantify the influence of residual autoblinking molecules, we analyzed the spatial distributions and diffusion coefficients of autoblinking and PprI-mMaple3 molecules. PprI-mMaple3 molecules tend to distribute in various regions of cells without clear bias, whereas autoblinking molecules localize preferentially on cell membrane. In addition, the total number of PprI-mMaple3 molecules was dominantly larger than autoblinking molecules acquired during the same acquisition time (Figure 4A), suggesting autoblinking molecules only contribute <6.5% molecule localizations to the recorded signals. Most of the autoblinking molecules can be removed from the collected signal by the above-mentioned filtering process of intensity and duration frames.

In living cells, PprI molecules may exist in different motion states, depending on their specific functionalities. Based on previous studies, it is known that most proteins are in two states of motion. Therefore, we first classified PprI molecules into two species and free-fitted to obtain a diffuse population with D* > 0.11 μm^2^/s and an immobile population with D* < 0.11 μm^2^/s (Stracy et al., 2021). In 32766 frames, we collected 37942 PprI-mMaple3 molecules in total, making up 2532 trajectories, of which diffusing molecules accounted for 89.8% of the total. A total of 1935 autoblinking molecules were observed, constituting 165 single-molecule trajectories, which equals 6.5% of that from PprI-mMaple3 (Figures 4B–D). Therefore, autoblinking signals cover only a tiny fraction of overall mMaple3 molecules, ∼5.1% for localizations and ∼6.5% for linked trajectories. For the categorized diffusing species, only 80 molecules were linked as trajectories, which equals ∼3.5% of that formed within diffusing PprI-mMaple3 molecules (Figure 4D), which is largely negligible for single-molecule imaging. Interestingly, we observed that more autoblinking molecules (1098/1935, ∼56.7%) stay as immobile state, while PprI-mMaple3 molecules tend largely to be the diffusing state (34066/37942, ∼89.8%). The trajectory lengths of PprI-mMaple3 molecules were significantly longer than those of autoblinking molecules, which can serve as another criterion for separating mMaple3 from autoblinking signals. The trajectory lengths of immobile molecules were slightly longer than those of diffusing molecules in both autoblinking and PprI-mMaple3 molecules (Figure 4E).

### 3.4. The localizations of PprI-mMaple3 molecules relative to the nucleoid

**FIGURE 5:**
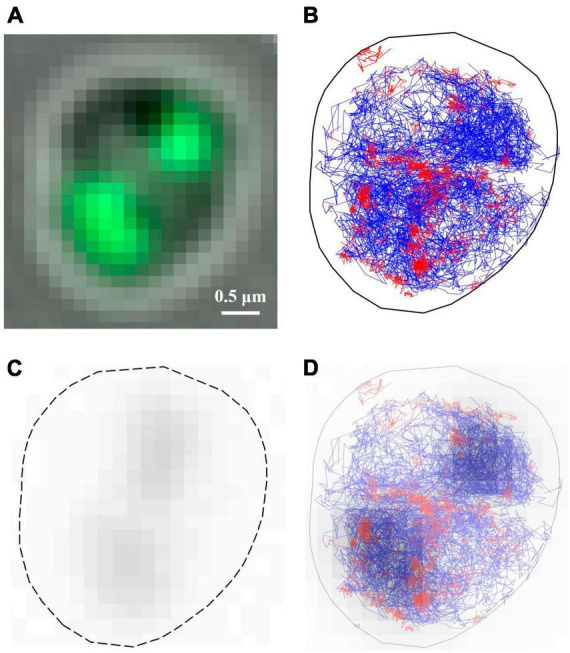
*Deinococcus radiodurans* nucleoid and distribution states of the PprI-mMaple3 protein in *D. radiodurans*. **(A)** Overlay of SYBR Gold-stained nucleoid with a bright-field plot of *D. radiodurans*. **(B)** Molecular trajectories of PprI (red: immobile molecules; blue: diffusing molecules). **(C)** Gray-scale plot of **(A)**. **(D)** Overlay of **(B,C)**. Scale bar: 0.5 μm in **(A)**.

**FIGURE 6:**
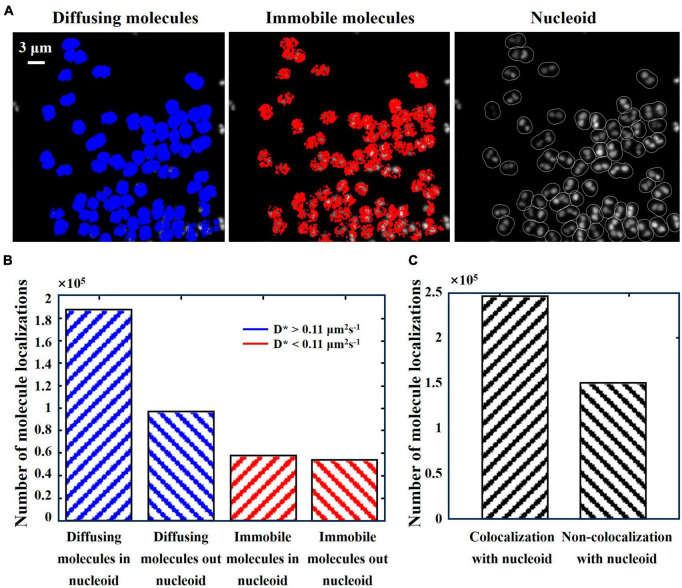
Relative positions of PprI and nucleoid. **(A)** Subcellular localization map of diffusing molecules (**left**, in blue) vs. immobilized molecules (**middle**, in red) in the same field of view and grayscale map of SYBR Gold-stained nucleoid (**right**). **(B)** Histogram of the number of diffusing and immobilized molecules with distribution inside and outside the nucleoid. **(C)** Histogram of the number of PprI molecules colocalized with and without the nucleoid. Scale bar: 3 μm in **(A)**.

We stained the nucleoid of *D. radiodurans* cells using SYBR Gold and acquired nucleoid fluorescence images under 488 nm laser irradiation (Figure 5A, green). Overlaying (Figure 5D) the molecular trajectories of Pprl-mMaple3 (Figure 5B) with the position of the nucleoid grayscale maps (Figure 5C), Matlab-based analysis yielded that the majority of Pprl molecules preferentially covered the nucleoid region, with diffusing molecules being more pronounced. In order to quantitatively analyze the relative positions of PprI proteins with respect to the nucleoid in *D. radiodurans* cells in the normal state, the PprI proteins were categorized into two types: immobilized molecules and diffusing molecules, according to the apparent diffusion coefficient, and the numbers of molecules inside and outside the nucleoid for these two motion states were calculated, respectively (Figure 6). It was found out that 65.6% of the diffusing molecules localized within the nucleoid, whereas the tendency of the immobilized molecules was not significant (Figure 6B). Taken together, a larger fraction of PprI molecules co-localized with the nucleoid, accounting for ∼62.1% of the total number of PprI molecules (Figure 6C).

### 3.5. Different motion states of PprI molecules in living *D. radiodurans* cells

**FIGURE 7:**
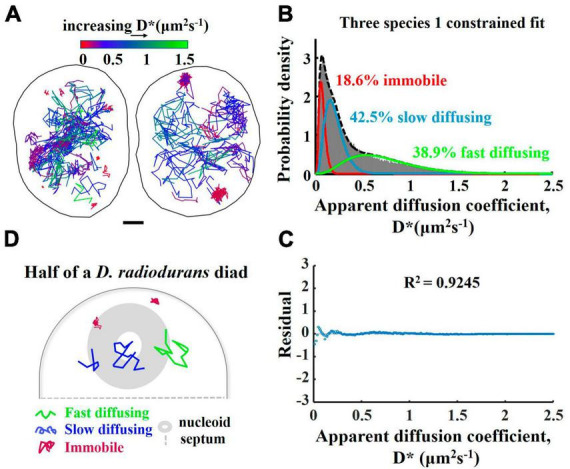
Motion behavior of the PprI-mMaple3 protein within the *Deinococcus radiodurans* cells. **(A)** Single-molecule trajectory map of PprI-mMaple3 in living *D. radiodurans* cells (**left:** diad cell; **right:** tetrad cell). **(B)** Apparent diffusion coefficient (D*) distribution of the 131,025 tracked molecules of PprI-mMaple3, fitted to three species. **(C)** Residual plot of the fitted curve for D* distribution in **(B)**. **(D)** Schematic representation of three PprI protein movement states in *D. radiodurans*.

To measure the mobility of PprI molecules in living *D. radiodurans*, we performed single-molecule tracking with DR-PprI-mMaple3 strain in MM (Figure 7A). We first calibrated the localization error of our microscope setup by measuring the mMaple3 molecules in fixed *E. coli* cells and obtained the averaged D* was ∼0.06 μm^2^/s and the localization error as ∼30 nm (Supplementary Figure 6). Instead of an inappropriate two-species fitting (Supplementary Figure 5), the D* distribution of PprI-mMaple3 molecules was fitted into a three-species model (Figure 7B) with *R*^2^ = 0.9245 (Figure 7C), shown as the immobile molecules (18.6%, D* = 0.07 μm^2^/s), slow diffusing molecules (42.5%, D* = 0.20 μm^2^/s), and fast diffusing molecules (38.9%, D* = 0.72 μm^2^/s) (Figure 7B). The residual plot shows the fitting is optimal (Figure 7C). From the color-coded trajectories of PprI molecules, we found that the majority (>80%) of PprI molecules belong to the fast and slow diffusing species, which tend to search freely for the target in the nucleoid-free regions and to bind transiently the target of DNA or protein partners within the nucleoid regions (Figure 7D). However, the immobile species is supposed to engage into active reaction with DNA or protein partners in the DNA repair pathway (Figure 7D).

## 4. Discussion

Based on the temporal distribution of background brightness, we found that both DR-WT and DR-PprI-mMaple3 exhibited extremely strong background noise in TGY, while incubation with PBS and MM allowed to greatly reduce the background (Figures 2A, B). In the spatial distribution of background intensity values (Figures 2C, D), the span in TGY is larger than that in PBS or MM, largely due to the unavoidable background noise, which proves again the reliability of PBS and MM imaging medium for background reduction. It has been found that the main source of the autoblinking phenomenon may be the cell growth medium, which causes autoblinking due to the transient binding of fluorescent molecules in the cell growth medium to the cell wall of bacteria (Floc’h et al., 2018). As a medium for fast-growing, TGY is enriched with a variety of nutrients such as numerous amino acids, which enhances the appearance of fluorescent molecules and therefore results in stronger autoblinking phenomenon. Compared to TGY, MM medium has a simpler nutrient composition and a smaller variety of amino acids (Supplementary Table 2), which may help suppress the autoblinking phenomenon to some extent. As for the reduction of fluorescent molecules in the imaging medium, washing steps by PBS solution for six times or the employment of MM in place of TGY enabled *D. radiodurans* bacteria to exhibit the lowest possible autoblinking phenomenon, which is consistent with the previous study (Floc’h et al., 2018), thereby providing favorable cellular imaging conditions for single-molecule tracking assay. The use of MM medium reduces the pre-treatment process for imaging bacteria, improves the reproducibility of experiments, and suppresses variations due to experimental technique.

It has been previously shown that autoblinking in bacteria is difficult to distinguish from PAmCherry (Liao et al., 2015; Tuson et al., 2016; Garza de Leon et al., 2017; Floc’h et al., 2018), leading to the fact that it becomes challenging to perform single-molecule tracking in bacteria containing strong autoblinking, especially for protein with low copy number and associated with cell wall (Tuson et al., 2016; Floc’h et al., 2018). In this study, we used mMaple3 (Baldering et al., 2019; Parmar and Weber, 2023), a brighter and faster maturation protein, in place of PAmCherry to label the PprI proteins. It was found that mMaple3 provided a much larger number of PprI localizations. A potential reason is the differences in the maturation time of these two fluorescent proteins. Because *D. radiodurans* has a relatively shorter doubling time compared to mammalian cells, a substantial fraction of the PAmCherry fluorescent protein molecules may not have sufficient time to mature and fluoresce. In comparison, mMaple3 has a faster maturation time rate PAmCherry, allowing us to acquire more molecules in the same imaging time window (Wang et al., 2014). The SBR of mMaple3 was significantly higher than that of autoblinking and PAmCherry (Supplementary Table 4). Autoblinking in TGY imaging medium was indistinguishable from mMaple3 under the same imaging conditions. And the use of PBS or MM imaging medium enhances the difference between mMaple3 and autoblinking due to the reduction of background and intensity of autoblinking. Thus, culture and preparation in MM medium can provide a favorable imaging medium for single-molecule tracking in live *D. radiodurans* cells. Considering the phenomenon of minimal autoblinking in MM, we found that the average frame duration for autoblinks is only 1.41 frames, mean squared displacement (MSD) analysis is used to extract reliable values of the diffusion coefficient of single-molecules. Short trajectories (e.g., <4 steps) generally introduce statistical uncertainties in MSD analysis and are usually discarded (Stracy et al., 2014). An apparent diffusion coefficient, D*, was calculated from the mean-squared displacement (MSD) for each track with a minimum of 4 steps (Stracy et al., 2015, 2016) to reduce the statistical uncertainty in the MSD values (Uphoff et al., 2014). Of course, greater than 4 frames will remove more autoblinking molecules, but also lose more PprI-mMaple3 molecules, so we balance with 4 frames as the threshold (Stracy et al., 2014). As a result, we were able to remove the vast majority of the collected autoblinking molecules according to the differences in the number of duration frames, further reducing the impact of autoblinking on the tracking of single-molecules.

The autoblinking and PprI-mMaple3 molecules localize at different subcellular positions inside the cells. We found that autoblinking molecules mainly distribute around the cell membrane (Figure 4A), which is in agreement with previous studies (Floc’h et al., 2019), and that the number of residual autoblinking molecules was very low upon our filtering strategy to remove most of the autoblinking molecules. PprI-mMaple3 molecules, on the other hand, mainly distribute in various nucleoid-proximal regions within the cell, with a number much larger than that of the autoblinking molecules. PprI-mMaple3 molecules display overall dispersed and clustered distribution with different diffusion coefficients, and we suppose that there may be both diffusing and immobile species of PprI, serving different biological functions. For both autoblinking and PprI-mMaple3 molecules, they are simply categorized into diffusing and immobile molecules. The localizations and trajectories of autoblinking molecules in both states are relatively shrunken, indicating that the residual autoblinking molecules post pre-bleaching and filtering steps are largely tolerable/negligible compared with that of PprI-mMaple3. We also notice that trajectory lengths of the immobile molecules were slightly longer than those of the diffusing molecules for both autoblinking and PprI-mMaple3 molecules, which may be due to that the diffusing molecules tend to travel throughout a large area with the possibilities out of the focal plane, whereas the immobile molecules located tightly at certain space around the focal plane can be acquired continuously till bleaching.

Through qualitative and quantitative methods, we found that under normal conditions, most of the diffusing PprI molecules in cells tend to the nucleoid region, while the localization tendency of immobilized PprI molecules is not obvious. In summary, PprI molecules are distributed throughout the cell, but more obviously tend to the nucleoid. The strong co-localization of diffusing Pprl molecules with the nucleoid suggests that the diffusing PprI molecules tend to search around the whole nucleoid region for the binding site, while the immobilized Pprl molecules interact with other protein factors beyond the nucleoid region and engage in active DNA repair related pathways.

It is worth noting that the frequency and magnitude of the laser are very important for single-molecule data acquisition. The power of the 405 nm laser affects the rate of activation of photoconvertible fluorescent proteins, and the optimal conditions for single-molecule tracking is to keep the number of activated molecules as one molecule in each cell per frame. Therefore, to start the activation with a low-power 405 nm laser, the number of mature photoconvertible fluorescent protein molecules to be bleached increases as the acquisition time grows, and the power of the 405 nm laser needs to be increased to meet the number of activated molecules. The 561 nm laser power affects the acquisition speed of the camera, the bleaching rate of the fluorescence signal, and the signal intensity. On the one hand, the higher the laser power of 561 nm, the higher the rate of fluorescence signal bleaching, and the corresponding acquisition speed of the camera is also faster until it reaches the maximum acquisition speed of the camera. On the other hand, the higher the 561 nm laser power, the higher the signal intensity of the fluorescent signal. The maximum acquisition frequency of the camera used in this study was 60 Hz. Therefore, in order to capture the entire trajectory of the fluorescence signal from activation to quenching as much as possible, a higher 561 nm laser power was used while maintaining the maximum acquisition frequency of the camera to maintain a better bleaching rate of the fluorescence signals as well as the signal intensity, so as to capture sharper and clearer molecules of the fluorescence signals.

Here we are able to perform single-molecule tracking of PprI protein in *D. radiodurans* bacteria, which is a big step forward compared with previous studies that have used PAmCherry-tagged HU proteins (Floc’h et al., 2018). However, previous work only performed single-molecule imaging for regional differentiation between cell wall-localized autoblinking and nucleoid-proximally localized HU proteins. In this study, we distinguished between autoblinking and PprI-mMaple3 through five aspects: intensity, duration frames, molecule number, trajectory lengths, and subcellular location. We demonstrate that the application of single-molecule tracking technology for PprI proteins can be achieved in *D. radiodurans* bacteria with autoblinking molecules using a process that optimization of experiments and analytical methods of data processing.

We have found a tendency for the majority of PprI diffusing molecules in cells in the normal state to prefer nucleoid, whereas the tendency for immobilized PprI molecules to localize is not obvious. We obtained the color-coded molecular trajectories of PprI molecules depending on the diffusion rates, of which the histogram was fitted into three species model. The actual D*_(immobile)_ = 0.07 μm^2^/s, which is slightly higher than the D* = 0.06 μm^2^/s obtained from the localization error, probably because, on the one hand, the D*_(immobile)_ is not entirely derived from the localization error, and PprI may also undergo conformational changes or movement on the DNA strand, etc. On the other hand, the localization error was calculated using the mMaple3 expressed by the plasmid in *E. coli*, which is slightly different from that of PprI-mMaple3 in *D. radiodurans*. We suppose that PprI protein exhibits three motional states, namely a fast-diffusing state in the cytoplasm, a slow-diffusing state with transient interactions with DNA, and an immobile state bound to DNA stably (Figure 7D).

In this article, we characterized in *D. radiodurans* the conditions of unavoidable autoblinking unaddressed in the previous study (Floc’h et al., 2018), and successfully applied single-molecule tracking assay of mMaple3-tagged PprI protein in order to gain nanoscale insights into its subcellular localization and dynamics. SMT is generally supposed to measure the diffusion rate of slow molecules in cells, such as the proteins on the cell membrane. Thus, when the target protein is moving slowly in bacteria, such as the PprI protein in this work, our strategy can work well to track the target protein at the single-molecule level by removing most of the autoblinking molecules. However, for fast-diffusing molecules, with an intracellular diffusion coefficient D of ∼20–30 μm^2^ s^–1^, the ∼10 ms frame time in typical wide-field single-molecule tracking experiments results in ∼700 nm of diffusion in each dimension, hence severe motion-blur and the pixel intensity of each true event as it appears in the image will fall. In this case, while SMT does not work well, people recently developed other single-molecule approach, for example single-molecule displacement/diffusivity mapping (SM*d*M) (Xiang et al., 2020) method, to measure the diffusion rate. SM*d*M reduces the temporal separation between captured image pairs by placing two excitation pulses at the end of the first frame and at the beginning of the second frame, thus allowing the tracking of fast-moving proteins. In other strains where the autoblinking phenomenon exists, our strategy provides a powerful means of studying the molecular mechanism of the proteins of interest or the dynamic real-time process of protein-protein interactions in other strains where the autoblinking phenomenon exists.

## 5. Conclusion

In bacteria with strong autoblinking phenomenon, such as in *D. radiodurans*, it was challenging to perform single-molecule tracking of target proteins of interest (Floc’h et al., 2018). We have demonstrated that it is feasible to carry out single-molecule tracking in *D. radiodurans* cells by eliminating and differentiating the originally high level of autoblinking molecules. In this study, we distinguish between autoblinking and fluorescent proteins (mMaple3) in bacteria in five ways that are essential for single-molecule tracking and super-resolution imaging in bacteria with autoblinking. With mMaple3 fused to PprI protein in the genome, the co-expressed proteins allow to minimize the interference of autoblinking in *D. radiodurans*. Removing the autoblinking molecules based on their unique photophysical characteristics (intensity and duration frames) enables us to unveil at nanometer resolution the details regarding the subcellular localizations and motion behavior of proteins of interest in living cells. Via the strategy in this article, single-molecule tracking assay can be applied for other bacteria and even some eukaryotic organisms with autoblinking phenomenon.

## Data availability statement

The original contributions presented in the study are included in the article/Supplementary material, further inquiries can be directed to the corresponding authors.

## Author contributions

FZ: Methodology, Validation, Writing – original draft, Writing – review and editing, Data curation, Formal analysis, Visualization. LH: Formal analysis, Methodology, Visualization, Writing – original draft, Software. XC: Formal analysis, Methodology, Visualization, Validation, Writing – review and editing. TJ: Methodology, Visualization, Conceptualization, Data curation, Writing – original draft. QG: Methodology, Visualization, Writing – original draft, Software. LX: Methodology, Software, Visualization, Writing – original draft. YM: Conceptualization, Resources, Writing – review and editing. XD: Conceptualization, Resources, Writing – review and editing. ZZ: Funding acquisition, Investigation, Supervision, Writing – original draft. KC: Funding acquisition, Investigation, Supervision, Writing – original draft, Conceptualization, Software, Writing – review and editing. JF: Conceptualization, Funding acquisition, Investigation, Software, Supervision, Writing – original draft, Writing – review and editing, Methodology, Project administration, Validation.
