# Supplementary material for: Single-molecule tracking of PprI in D. radiodurans without interference of autoblinking
Source: Front Microbiol. 2023 Nov 2;14:1256711. doi: 10.3389/fmicb.2023.1256711 (PMC10652783; doi:10.3389/fmicb.2023.1256711)
Supplement: Supplementary file 8 [file Data_Sheet_1.docx]

Supplementary Material

**Single-molecule tracking of PprI in *D. radiodurans* without interference of autoblinking**

Fanfan Zhai^1, 2, 3†^, Li Hao^2†^, Xiaomin Chen^2^, Ting Jiang^2^, Qianhong Guo^1, 2^, Liping Xie^1, 2^, Ying Ma^3^, Xiaobo Du^3^, Zhiqin Zheng^1, 3, 4*^, Kun Chen^5, 6*^, Jun Fan^2, 6*^

*** Correspondence:**Zhiqin Zheng, Kun Chen, Jun Fan
zhengzhiqin168@163.com or chenk21@uestc.edu.cn or [fan.jun@uestc.edu.cn](mailto:fan.jun@uestc.edu.cn)

# Supplementary Figures

**Supplementary Figure 1.** Five background locations of 4×4 pixels with no cells were selected from one video of 256×256 pixels in the TGY, PBS, and MM environments for both wild type (DR-WT, without introducing any extrinsic fluorescent markers) and fluorescently labeled *D. radiodurans* (DR-PprI-mMaple3), respectively. Five background locations of 4×4 pixels of DR-WT in TGY **(A)**, PBS **(B)**, and MM **(C)** imaging medium. Five background locations of 4×4 pixels of DR-PprI-mMaple3 in TGY **(D)**, PBS **(E)**, and MM **(F)** imaging medium. Scale bar: 5 μm (**A-F** raw data), 0.16 μm (**A-F** subimages).

**Supplementary Figure 2.** Background areas of 4 regions of 50 × 50 pixels of DR-WT **(A-C)** and DR-PprI-mMaple3 **(D-F)** in TGY, PBS and MM imaging medium. Scale bar: 5 μm in **(A-F)**.

**Supplementary Figure 3.** Fluorescence signals of DR-WT and DR-PprI-mMaple3 strains in TGY, PBS and MM imaging environments. A fluorescence signal of 4×4 pixels was selected from one video of 256×256 pixels in the TGY **(A)**, PBS **(B)**, and MM **(C)** environments for DR-WT bacterial. A fluorescence signal of 4×4 pixels was selected from one video of 256×256 pixels in the TGY **(A)**, PBS **(B)**, and MM **(C)** environments for DR-PprI-mMaple3 bacterial. Scale bar: 5 μm (**A-F** raw data), 0.16 μm (**A-F** subimages).

**Supplementary Figure 4.** Background intensity of DR-WT and DR-PprI-mMaple3 in four regions in TGY, PBS and MM environments. **(A-C)** Histogram of background intensity of DR-WT bacteria in TGY, PBS, MM medium at 4 regions of 50 × 50 pixels. **(D-F)** Histogram of background intensity of DR-PprI-mMaple3 bacteria in TGY, PBS, MM medium at 4 regions of 50 × 50 pixels.

**Supplementary Figure 5.** Two-species fits for PprI-mMaple3. The significant deviations from of the fits from the D* distributions show that a two-fit cannot describe the D* distribution well. Fixing the rate in the immobile state at 0.07µm^2^/s and the rate in the diffuse state free.

**Supplementary Figure 6.** Localization error obtained by measuring 100 mMaple3 molecules in fixed *E. coli* cells. Each molecule gives a cluster of localizations owing to continuous acquisition of the same molecule. Localizations from 100 clusters (each containing >8 localizations) are aligned by their center of mass to generate the 2D presentation of the localization distribution. Histograms of distribution in x and y are fit to Gaussian functions, and the resultant s.d. (σ_x_ and σ_y_) is shown.

**Supplementary Figure 7.** Experimental flow chart.

# Supplementary Tables

**Supplementary Table 1.** Primers used in this study

| Name | Description |
| --- | --- |
| *pprI* (pCE-Zero) For | AATTCGGATCTTCCAGAGATAACGTCAGCCCCCCTTGC |
| *pprI* (mMaple3) Rev | GAACCAGCAGCAGAACCAGCGCTCTGTGCAGCGTCCTGCGG |
| mMaple3 (*pprI*) For | CCGCAGGACGCTGCACAGAGCGCTGGTTCTGCTGCTGGTTC |
| mMaple3 (Kan) Rev | ATCAAGCTTATCGATACCGTTTACTTGTACAGCTCGTCCA |
| Kan (mMaple3) For | TGGACGAGCTGTACAAGTAAACGGTATCGATAAGCTTGAT |
| Kan (*folp*) Rev | TGGATACGGCCCGTCCAGTTCACTAGAGGATCCTGATTAGAAA |
| *folp* (Kan) For | TTTCTAATCAGGATCCTCTAGTGAACTGGACGGGCCGTATCCA |
| *folp* (pCE-Zero) Rev | TTCAACTGCCGTTCGACGATCGCCGCCTCCACCCGCAG |
| *PprI*For | AACGTCAGCCCCCCTTGC |
| *folp*Rev | CGCCGCCTCCACCCGCAG |
| *pprI* (upstream) For | TTCGAGTCCCGCGAGGGGAG |
| *folp* (downstream) Rev | GTGCAGTTCGGCGTCCACGA |
| pCE-ZeroM13For | CAGGAAACAGCTATGAC |
| pCE-ZeroM13Rev | GTAAAACGACGGCCAGT |
| pET-28a-mMaple3 (EcoRI) For | CCGgaattcTCTGCTGGTTCTGCTGCTGG |
| pET-28a-mMaple3 (HindIII) Rev | CCCaagcttTTACTTGTACAGCTCGTCCATGCT |
| pCE-Zero-Vector | Linearized (ClonExpress® Ultra One Step Cloning Kit) |
| pET-28a-mMaple3 | Full length mMaple3 cloned into pET-28a using EcoRI and HindIII cloning sites |

Note: Kan: kanamycin resistance gene; Underlined are homologous sequences; Lowercase letters represent enzyme cutting sites

**Supplementary Table 2.** Complete recipe of minimal medium (MM)

| Component | Concentration necessary for growth |
| --- | --- |
| Potassium phosphate buffer (PH 7.6) | 20 mM |
| Magnesium chloride hexahydrate | 0.2 mM |
| Calcium chloride | 0.1 mM |
| Manganese (II) acetate tetrahydrate | 5.0 μM |
| Ammonium molybdate tetrahydrate | 5.0 μM |
| Ferrous sulfate heptahydrate | 5.0 μM |
| L-Histidine | 25 μg/ml |
| L-Cysteine | 30 μg/ml |
| Nicotinic acid | 1.0 μg/ml |
| Fructose | 2.0 mg/ml |

**Supplementary Table 3.** The SBR and intensity of autoblinking and mMaple3 molecules

| Strain | Media | Fig. 3 **A** and **B** | | | Fig. 3 **E** and **F** | |  |
| --- | --- | --- | --- | --- | --- | --- | --- |
|  |  | Background-bacterial | Peak Intensity | SBR | Signal Intensity | Standard Deviation (SD) | |
| DR-WT (Autoblinking) | TGY | 289 | 579 | 2.00 | 572 | 70.40 | |
|  | PBS | 146 | 289 | 1.98 | 372 | 61.70 | |
|  | MM | 185 | 379 | 2.05 | 382 | 45.02 | |
| DR-PprI-mMaple3 | TGY | 250 | 740 | 2.96 | 664 | 160.45 | |
|  | PBS | 171 | 601 | 3.51 | 606 | 117.32 | |
|  | MM | 182 | 652 | 3.58 | 661 | 117.87 | |

**Supplementary Table 4.** Comparison of the performance between PprI-mMaple3 and PprI-PAmCherry

|  | Signal-to-Background Ratios (SBR) | | | | | | | | SBR mean | Number of localizations (14654 frames) |
| --- | --- | --- | --- | --- | --- | --- | --- | --- | --- | --- |
| Molecule | 1 | 2 | 3 | 4 | 5 | 6 | 7 | 8 |  |  |
| PprI-mMaple3 | 2.64 | 2.16 | 2.14 | 3.97 | 2.44 | 2.14 | 2.16 | 3.56 | 2.65 | 548 |
| PprI-PAmCherry | 1.77 | 1.75 | 1.86 | 2.28 | 2.05 | 2.09 | 2.24 | 1.84 | 1.98 | 133 |
| Autoblinking | 1.64 | 1.76 | 1.67 | 1.88 | 1.90 | 1.97 | 1.75 | 1.63 | 1.77 | --- |

# Supplementary Video

**Supplementary Video 1.** Single-molecule tracking PALM imaging of DR-WT bacteria in TGY. Wild-type *D. radiodurans* bacteria were cultured and imaged in TGY medium and pre-bleached using high power (23.0 mW) 561 nm excitation light prior to data collection. Imaging conditions were consistent with fluorescently labelled bacteria using low 561 nm excitation power (2.7 mW) and low 405 nm activation power (1.48-3.69 µW). 0.01746 s per image frame, 300 frames in video, Scale bar, 5 μm.

**Supplementary Video 2.** Single-molecule tracking PALM imaging of DR-WT bacteria in PBS. Wild-type bacteria were cultured in TGY medium, washed six times with PBS buffer to remove as much of the TGY medium as possible before imaging, and imaged in PBS medium. Imaging conditions were identical to those for DR-WT imaging in TGY (Supplemental video 1). 0.01746 s per image frame, 300 frames in video, Scale bar, 5 μm.

**Supplementary Video 3.** Single-molecule tracking PALM imaging of DR-WT bacteria in MM. Wild-type bacteria are first activated in TGY medium, then transferred to MM medium for expanded culture and imaging. Imaging conditions were identical to those for DR-WT imaging in TGY (Supplemental video 1). 0.01746 s per image frame, 300 frames in video, Scale bar, 5 μm.

**Supplementary Video 4.** Single-molecule tracking PALM imaging of DR-PprI-mMaple3 in TGY. *D. radiodurans* Strains with PprI protein in situ tagged with mMaple3 were cultured and imaged in TGY medium. Imaging conditions were identical to those for DR-WT imaging in TGY (Supplemental video 1). 0.01746 s per image frame, 300 frames in video, Scale bar, 5 μm.

**Supplementary Video 5.** Single-molecule tracking PALM imaging of DR-PprI-mMaple3 in PBS. DR-PprI-mMaple3 bacteria were cultured in TGY medium, washed six times with PBS buffer to remove as much of the TGY medium as possible before imaging, and imaged in PBS medium. Imaging conditions were identical to those for DR-WT imaging in TGY (Supplemental video 1). 0.01746 s per image frame, 300 frames in video, Scale bar, 5 μm.

**Supplementary Video 6.** Single-molecule tracking PALM imaging of DR-PprI-mMaple3 in MM. DR-PprI-mMaple3 bacteria are first activated in TGY medium, then transferred to MM medium for expanded culture and imaging. Imaging conditions were identical to those for DR-WT imaging in TGY (Supplemental video 1). 0.01746 s per image frame, 300 frames in video, Scale bar, 5 μm.

**Supplementary Video 7.** Single-molecule tracking PALM imaging of DR-PprI-PAmCherry in MM. *D. radiodurans* Strains with PprI protein in situ tagged with PAmCherry are first activated in TGY medium, then transferred to MM medium for expanded culture and imaging. Imaging conditions were identical to those for DR-WT imaging in TGY (Supplemental video 1). 0.01746 s per image frame, 300 frames in video, Scale bar, 5 μm.
